# Quantification of fixed adherent cells using a strong enhancer of the fluorescence of DNA dyes

**DOI:** 10.1038/s41598-019-45217-9

**Published:** 2019-06-18

**Authors:** Anna Ligasová, Karel Koberna

**Affiliations:** Institute of Molecular and Translational Medicine, Faculty of Medicine and Dentistry, Palacký University in Olomouc, Olomouc, 779 00 Czech Republic

**Keywords:** Cell growth, High-throughput screening

## Abstract

Cell quantification is widely used in basic or applied research. The current sensitive methods of cell quantification are exclusively based on the analysis of non-fixed cells and do not allow the simultaneous detection of various cellular components. A fast, sensitive and cheap method of the quantification of fixed adherent cells is described here. It is based on the incubation of DAPI- or Hoechst 33342-stained cells in a solution containing SDS. The presence of SDS results in the quick de-staining of DNA and simultaneously, in an up-to-1,000-fold increase of the fluorescence intensity of the used dyes. This increase can be attributed to the micelle formation of SDS. The method is sufficiently sensitive to reveal around 50–70 human diploid cells. It is compatible with immunocytochemical detections, the detection of DNA replication and cell cycle analysis by image cytometry. The procedure was successfully tested for the analysis of cytotoxicity. The method is suitable for the quantification of cells exhibiting low metabolic activity including senescent cells. The developed procedure provides high linearity and the signal is high for at least 20 days at room temperature. Only around 90 to 120 minutes is required for the procedure’s completion.

## Introduction

Cell quantification is a common task for many laboratories. A typical example of its use is drug-discovery research. Presently, several methods are available. They are usually based on the time-consuming direct calculation of cells using e.g. a haemocytometer or a much easier determination of the relative cell concentrations. Since the determination of the relative concentrations of cells is sufficient in many studies, these methods are a common tool for routine cell quantification. In addition, if necessary, the absolute number of cells can be determined after the calibration of the signal using samples containing a known number of cells.

Several strategies were developed for the determination of the relative number of cells. A very common strategy is based on the conversion of various substrates by cellular enzymes followed by the measurement of the concentration of the reaction’s product. Typical examples are tetrazolium-based assays and the methods based on Alamar Blue^1^. Tetrazolium-based assays are based on the reduction of the tetrazolium salts to the purple formazan crystals by mitochondrial dehydrogenases^2,3^. The most commonly used tetrazolium compound is (3-(4,5-dimethylthiazol-2-yl)-2,5-diphenyltetrazolium bromide (MTT) which was originally developed to measure cell proliferation and cytotoxicity^1,4^. As the reduction of MTT leads to the creation of insoluble formazan crystals which have to be solubilised with e.g. DMSO, other tetrazolium-based assays such as 3-(4,5-dimethylthiazol-2-yl)-5-(3-carboxymethoxyphenyl)-2-(4-sulfophenyl)-2H-tetrazolium (MTS) and 4-(3-(4-iodophenyl)-2-(4-nitrophenyl)2H-5-tetrazolio)-1,3-benzene disulphonate (WST-1) were developed^1,4^. In these assays, the water-soluble formazan products are formed and therefore, no solubilisation step is needed^4–7^. Alamar blue is one of the colorimetric assays as well. It is based on the conversion of the blue resazurin to the highly fluorescent pink resorufin by mitochondrial enzymes^8,9^. The disadvantage of the mentioned methods is their dependence on the metabolic state of the cell population. As the metabolic state can be related also to the density of the cell population^10^, it commonly results in the non-linear dependence of the signal on the cell number. The dependence on the metabolic state can also result in low sensitivity if the metabolically less active cells, e.g. senescent cells, are quantified. Moreover, the specific conditions have to be found for the individual cell lines.

The dependence on the metabolic state can be overcome by using methods based on the detection of cellular DNA. Fluorescent substances, which bind to DNA are examples. Their binding to DNA is accompanied by the significant increase of their fluorescence. This group includes cyanine dyes such as CyQuant, PicoGreen or SYBR Green I^11–13^. Other examples are DAPI^14,15^ or Hoechst stains^13,14^. The methods based on DNA staining do not depend on the cell metabolism and some of them exhibit sufficient sensitivity to reveal several tens of cells. However, cell lysis is usually required for maximal sensitivity and the linearity of the dependence of the signal on the cell number. It can result in the substantial prolongation of the procedure and/or additional costs. In addition, the stability of the signal is usually low and requires relatively fast evaluation of the processed cells or freezing of the sample.

In the study presented, we have developed an approach for the quantification of the fixed cells that does not require cell lysis as the DNA dye is eluted from cellular DNA using an elution solution. It results in a highly homogeneous solution of the eluted DNA dye and substantial increase of its fluorescence intensity. Therefore, only a fraction of the sample can be measured without any decrease in the accuracy of the measurement. As there is no need for cell lysis, the cell cycle analysis by image cytometry or the detection of various cellular components can be performed before the elution step. Moreover, all the components are cheap. It provides the possibility to quantify the cell number in various vessels including well plates, cultivation flasks, Petri dishes or even coverslips at reasonable cost.

## Results

### Method description

#### Method overview

**Figure 1:**
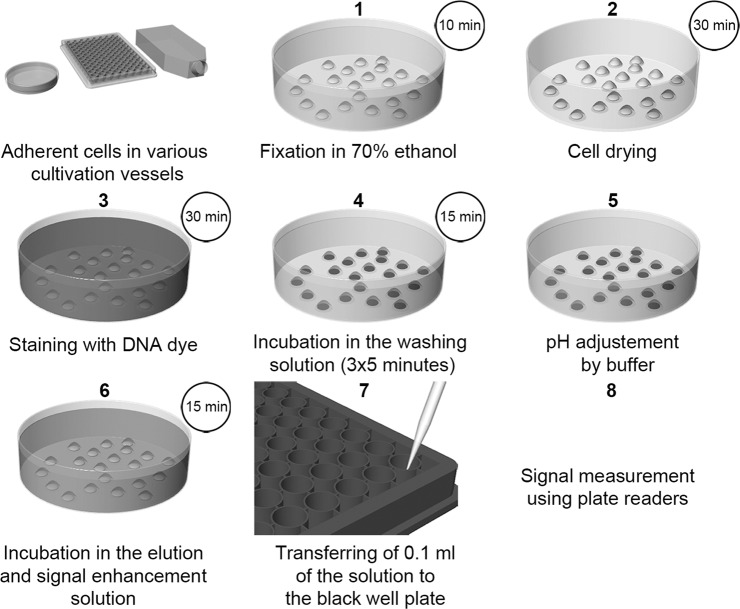
The scheme of the method. The scheme of the procedure with the recommended incubation times for Hoechst staining is shown. If DAPI is used instead of Hoechst dyes, the incubation in step 4 (the washing step) should be shortened to 3 × 2 minutes. The other incubation times are the same as in the case of Hoechst dyes. Step 2 (cell drying) is optional, but it is highly recommended if 96-well plates are used.

The scheme of the method is shown in Fig. 1. Adherent cells cultivated in various vessels are fixed with 70% ethanol for 10 minutes at room temperature (RT) and air-dried for 30 minutes. Subsequently, the cells are incubated in a solution of 2 µM Hoechst 33342 or 3 µM DAPI in 20 mM Tris-HCl, pH 7, 150 mM NaCl for 30 minutes on a laboratory shaker (100 µl per well of a 96-well plate). Afterwards, the cells are washed in a washing solution containing 2 mM CuSO_4_, 0.2 M CaCl_2_, 2 M NaCl, 0.2% Tween 20 and 50 mM citric acid for 5 minutes (Hoechst-stained cells) or in 2 mM CuSO_4_, 0.5 M NaCl, 0.2% Tween 20 and 20 mM citrate buffer, pH 5 for 2 minutes (DAPI-stained cells; 200 µl per well of a 96-well plate). This step is repeated three times in total and serves to remove the non-specifically bound dye and also to stabilise the cells. This stabilisation protects the cells from lysis when incubated in the elution buffer. Then, cells are briefly rinsed with the buffer to adjust a pH to the value around 7. In this respect, a 20 mM phosphate buffer, pH 7 with a 150 mM NaCl or 20 mM Tris-HCl buffer, pH 7 with 150 mM NaCl are used for Hoechst- or DAPI-stained cells, respectively. In the final step, the dye bound to DNA is eluted using the elution solution for 15 minutes on the laboratory shaker (120 µl per well of a 96-well plate). The elution solution contains 2% sodium dodecyl sulphate (SDS) and a 20 mM phosphate buffer, pH 7 or a 20 mM Tris-HCl, pH 7, for Hoechst- or DAPI–stained cells, respectively. The elution buffer serves for the elution of the dyes from the DNA and the signal enhancement. The aliquots of the elution solution are transferred to the black well plate (100 µl per well of a 96-well plate). The measurement is performed using the plate reader. The excitation wavelength should be 370 nm. The emission wavelength should be 460 nm in the case of the DAPI and 485 nm in the case of the Hoechst dye. For the background correction, the procedure is done in the culture vessel without cells. Its value is then subtracted from the value of the tested sample(s). Although Hoechst 33258 was tested as well, this dye provided less reliable data than Hoechst 33342 (see below). If coverslips with the diameter up to 15 mm are used, most steps can be performed on drops of solutions and elution can be performed in a 24-well plate.

#### Fixation

We tested several fixation protocols. We compared the signal in samples fixed with 70% ethanol or 100% methanol at various temperatures (−20 °C, 0 °C or RT) and for various times (10 minutes, 30 minutes, 1 hour or overnight), fixation by air-drying and formaldehyde fixation. With the exception of formaldehyde fixation, the rest of the fixation protocols used exhibited similar signals. The 10-minute fixation with 2% formaldehyde provided around half of the signal obtained by the ethanol or methanol fixation or by air-drying. As the 10-minute fixation with 70% ethanol at RT resulted in the effective protection of the cells from loosening from the cultivation surfaces and ethanol is less toxic than methanol, we used ethanol fixation in all the subsequent experiments. In contrast to the formaldehyde fixation, the ethanol fixation enables interruption of the procedure for a relatively long time. In this respect, the storage of samples for 3 months in 70% ethanol at −20 °C was successfully tested without any impact on the signal intensity. Therefore, if necessary, the procedure can be interrupted for several months after ethanol fixation.

Drying of the cells is performed immediately after ethanol fixation. This step is optional; however, it is highly recommended if the cells are incubated in 96-well plates. It allows stronger attachment of the cells to the cultivation surface and protects the cells from loosening during the washing steps as the repeated fluid stream from the pipette tip can release a substantial portion of the cells from the well. The cell drying after ethanol aspiration typically lasts 20 to 30 minutes depending on the temperature, humidity and the amount of ethanol volume resting in the well. It can be easily controlled as ethanol remnants are visible at the bottom of the well plates. This step is typically useless if plates with larger wells or Petri dishes or culture flasks are used. The cell drying should not exceed one hour progressively as extensive drying can result in the necessity of prolonging the elution step.

#### Cell staining

200 µl of the 2–3 µM dye solution is sufficient for staining cells grown on 1 cm^2^ of the cultivation area. In the case of 96-well plates, we used 100 µl of 3 µM DAPI or 2 µM Hoechst dye per well. If a Petri dish with the diameter of 3.5 cm was used, 2 ml of the dye solution was added. The additional increase of the dye concentration can result in the non-specific binding of the dye to the surface of the culture vessels and also to the non-linear dependence of the signal on the cell number. On the other hand, the lower concentrations can result in a lower signal per cell if a high density of cells is reached due to the depletion of a substantial portion of the dye from the solution. Thirty minutes is the optimal time with respect to the speed and the signal intensity. However, longer incubation times can be used without any effect on the results. On the contrary, the shorter times can result in a lower signal and lower sensitivity. The samples should be shaken, for example at 300 rpm, during the incubation, as the shaking accelerates the binding of the dye to the DNA.

**Figure 2:**
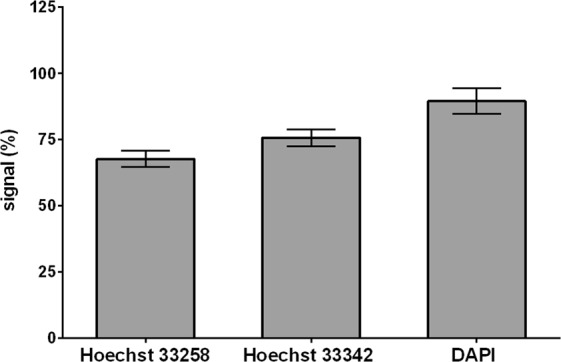
The impact of RNase A on the signal. The impact of RNase A on the signal of Hoechst 33258 or Hoechst 33342 or DAPI is shown. The cells were fixed by ethanol, dried and incubated in 1× PBS with or without RNase A for 1 hour at 37 °C and the developed approach was used. The signal is normalised to the signal measured in samples without RNase A treatment which is equal to 100%. The data are presented as the mean ± SD.

Although it is generally supposed that Hoechst dyes bind preferentially to DNA, it was also shown that they may bind to RNA as well^16,17^. Similarly, DAPI is supposed to bind to both DNA and RNA^18^. In order to estimate the contribution of RNA-bound dyes to the overall signal intensity we analysed the signal dependence on the RNA content. In this respect, the RNase A treatment resulted in ca 33% decrease of the signal in the case of Hoechst 33258, ca 25% decrease of the signal if we used Hoechst 33342 and ca 10% decrease of the signal of DAPI comparing to the non-treated cells (Fig. 2).

#### Washing and cell stabilization

The washing is the crucial step. During its course, the non-specifically bound dyes are removed. Dyes can be non-specifically bound to the cells or to the surfaces of the culture vessels. According to our results, the surfaces of the common polystyrene cultivation vessels bind all tested DNA dyes. These non-specifically bound dyes have to be removed before the elution step, otherwise it will result in a lowering of the sensitivity of the approach. As a 40-minute washing time is usually necessary to remove a substantial portion of the DNA dyes from the culture surfaces using 1× PBS, we tested other solutions to accelerate this step.

In the case of Hoechst dyes, we found that the use of solutions with a low pH and relatively high ionic strength results in the very fast dissociation of the dyes from the cultivation surfaces. In this respect, the solution of 50 mM citric acid supplemented with 200 mM CaCl_2_ and 0.2% Tween 20 makes it possible to wash out nearly all the surface-bound Hoechst molecules in several minutes. In this respect, three washing steps each for five minutes are sufficient for the removal of the majority of the non-specific signal. Importantly, the prolongation of the washing time did not result in the lowering of the DNA-specific signal of Hoechst dyes and, therefore, this step can be prolonged according to the user needs. Additional tests have shown that the linearity of the signal dependence on the cell number can be significantly improved by the addition of 2 M NaCl to the washing solution. It is probably a result of the washing out of the non-specifically bound dye from the cellular components.

In the case of DAPI, we successfully tested a solution of 20 mM citrate buffer, pH 5 supplemented with 0.5 M NaCl and 0.2% Tween 20. As DAPI is partially removed also from cellular DNA in the case of low pH and the high ionic strength, the total time of the washing step should not exceed 15 minutes, otherwise, it can result in the progressive lowering of the signal.

In both cases, the washing solution contained 2 mM CuSO_4_ as well. The addition of copper sulphate efficiently protects the ethanol-, methanol-fixed cells and also air-dried cells from SDS-induced lysis. The cell lysis is accompanied by the progressive increase of the solution viscosity and difficulties with the transfer of the solution of the eluted dyes to the black well plates. No such treatment is necessary if formaldehyde-fixed cells are used. Although the incubation with copper ions can be performed at any time before the elution step, the addition of copper ions into the washing solution is advantageous as it does not result in the prolongation of the procedure.

**Figure 3:**
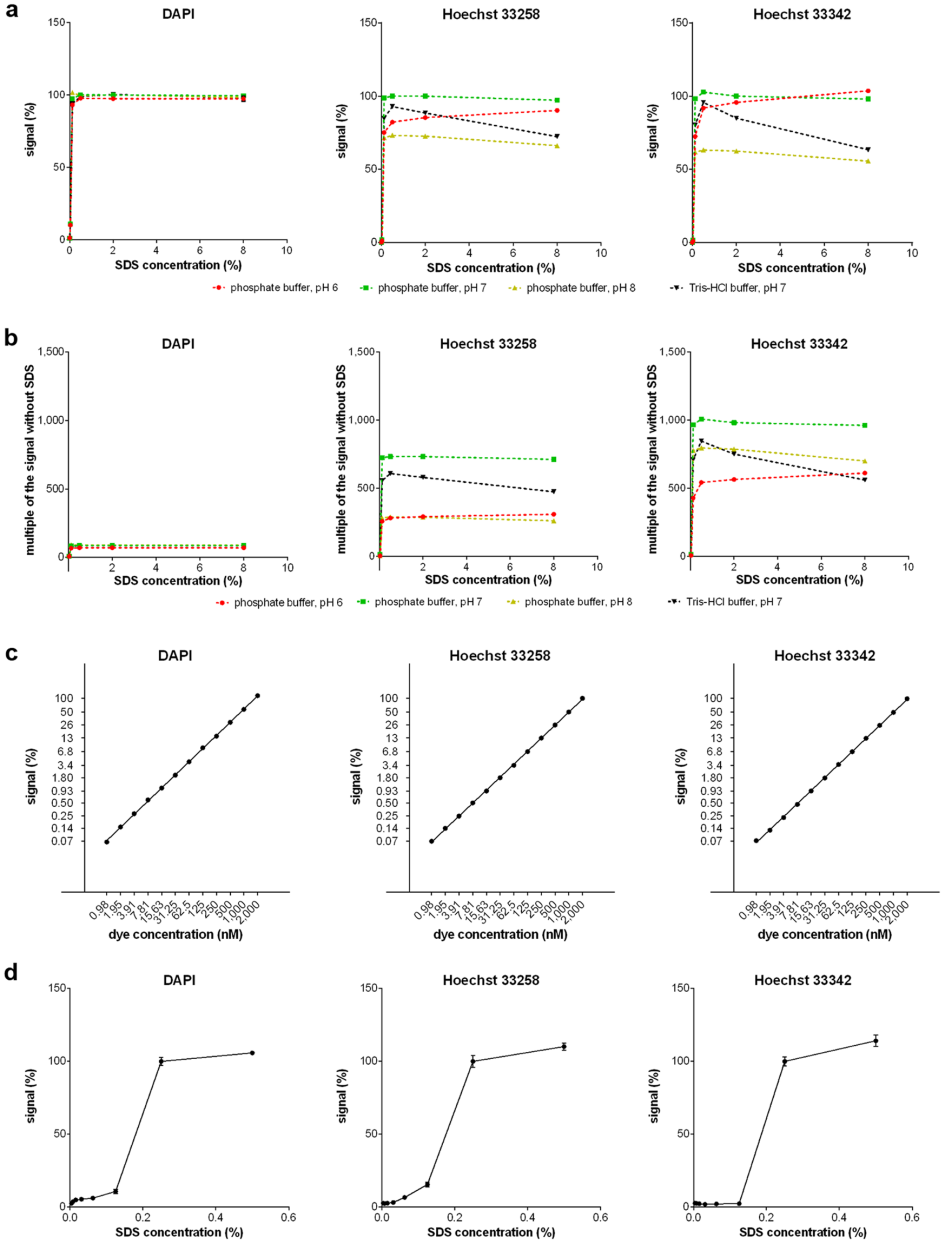
The dependence of the signal on the SDS and dye concentrations. (**a**) The dependence of the signal of the 1 µM solution of DAPI or Hoechst dyes on the percentage concentration of SDS in various buffers is shown. The signal is normalised to the signal measured in the samples with the DNA dye diluted in 2% SDS and a phosphate buffer, pH 7 which is equal to 100%. The data are presented as the mean ± SD. (**b**) The dependence of the signal enhancement of DNA dyes on the SDS concentration in various buffers is shown. The values of enhancement are expressed as a multiple of the signal intensity of the sample without the addition of SDS. The data are presented as the mean ± SD. (**c**) The dependence of the signal on the concentration of DNA dyes in 2% SDS is shown. In the case of DAPI, SDS was diluted in 20 mM Tris, pH 7, in the case of Hoechst dyes, SDS was diluted in 20 mM phosphate buffer, pH 7. The log scales of both dye concentrations and signal intensity are used. (**d**) The dependence of the signal of 1-µM solution of DAPI or Hoechst dyes on the percentage concentration of SDS in water is shown. The signal is normalised to the signal measured in samples with DNA dye diluted in 0.25% SDS in water which is equal to 100%. The data are presented as the mean ± SD.

After the washing step, the samples are rinsed with the solution of 20 mM phosphate buffer, pH 7 (if Hoechst dyes are used) or the Tris-HCl buffer, pH 7 (if DAPI is used) supplemented with 150 mM NaCl. The rinsing of the samples neutralizes the acid environment before the elution of the dyes from the DNA. This step is much more important in the case of Hoechst dyes as their signal highly depends on the pH value and the buffer composition (Fig. 3a). Samples can be left in the rinsing solution for several hours before proceeding to the elution step.

#### Elution

During the elution step, the samples are incubated in an aqueous, buffered solution of anionic detergent - SDS. Alternatively, lithium dodecyl sulphate can be used. This step assures the elution of the DNA dyes bound to the DNA in the solution.

It was previously shown that the presence of SDS in solutions leads to an increase of the fluorescence of DAPI and Hoechst 33258 and Hoechst 33342. This increase is attributed to micelle formation^19,20^. It is supposed that dyes enter the micelles which have a less polar inner core and this environment blocks the quenching of fluorescence by intramolecular proton transfer^20^. It was, nevertheless, necessary to determine at which concentration of SDS the signal will be the highest, whether it will be sufficient for the precise determination of the relative cell number, whether there is a cross-section between the concentration, which is necessary for the elution and the concentration for the sufficient increase of the signal and how linear the dependence of the signal is on the cell number in a sample. Optimally, the maximal fluorescent signal overlaps with values, which are necessary for the elution of dyes from the DNA of the samples and at the same time the fluorescent signal linearly increases with the increasing concentration of the dye and cells.

According to our results, the minimal concentration leading to the elution of the DNA dyes, was 0.1% SDS. However, this concentration required more than one-hour incubation for the effective elution of the dye from the DNA. In this respect, a solution of 2% SDS resulted in the effective elution of the DNA dyes in less than 15 minutes.

The analysis of the signal intensity of 1 µM dye solutions in buffers with different pH and various SDS concentrations showed a relatively high difference between the dyes used (Fig. 3a). In the case of Hoechst dyes, the signal intensity significantly depended on the buffer composition and pH. No such dependence was observed in the case of DAPI. The intensity of the Hoechst dyes was the highest in a phosphate buffer with pH 7 (Fig. 3a). The course of the signal dependence on SDS concentration in the case of Hoechst dyes in phosphate buffer, pH = 7 and DAPI in all the tested buffers showed that the signal intensity is similar in a wide range of SDS concentrations including the concentration suitable for the elution of both Hoechst dyes or DAPI from DNA.

The same experiment showed that the addition of SDS resulted up to 1,000-fold increase of the fluorescence intensity in the case of Hoechst 33342 and more than 700-fold increase in the case of Hoechst 33258 (Fig. 3b). Only an approximately 90-fold increase of the signal was measured if DAPI was used.

The dependence of the signal of both Hoechst dyes and DAPI in the elution solution containing 2% SDS on the dye concentration was linear in all the tested concentrations (Fig. 3c).

The comparison of the fluorescence intensity of the dyes in 2% SDS and 20 mM phosphate buffer, pH 7 (Hoechst dyes) or 20 mM Tris-HCl buffer, pH 7 (DAPI) showed that the highest fluorescence intensity was exhibited by Hoechst 33342. The fluorescence intensity of Hoechst 33258 was around 75% of the intensity of Hoechst 33342. The fluorescence intensity provided by DAPI was only around 45% of the intensity provided by Hoechst 33342 (Supplementary Fig. S1).

The dependence of the signal on the micelle formation was confirmed by the analysis of the dependence of the signal on the SDS concentration in the water (Fig. 3d). The CMC (critical micelle concentration – a concentration of the surfactant above which micelles are formed) of SDS in water is approximately equal to 8.1–8.4 mM or around 0.24%^21^.

To accelerate the elution, the samples should be agitated. Agitation of the samples at 300 rpm and RT is sufficient for the elution of DNA dyes from DNA in 15 minutes. Concerning the volume of elution solution, 120 µl per well of a 96-well plate and 0.5 ml per Petri dish with a diameter of 3.5 cm was successfully tested. After the completion of the elution step, 100 µl of the elution buffer containing the eluted DNA dye was carefully transferred to the black 96-well plates to maximize the signal/noise ratio and the signal was measured using the plate reader. The formation of bubbles should be avoided during the transfer as it can result in false results.

The signal can be measured immediately after the transfer of the solution to the black well plate or, if necessary, the black well plate can be sealed by parafilm and stored at RT until the measurement is performed. In this respect, the tested samples provided high fluorescent signal for at least twenty days (Supplementary Fig. S2).

We measured the excitation and emission spectra of DAPI in 20 mM Tris-HCl buffer, pH 7 with 2% SDS and Hoechst 33342 and Hoechst 33258 in 20 mM phosphate buffer, pH 7 with 2% SDS. The obtained data showed that the excitation spectra are similar in all the tested DNA dyes. In this respect, the maximum fluorescence intensity can be measured at an excitation around 370 nm. Concerning the emission spectra, a difference was observed between DAPI and Hoechst dyes. The highest emission was recorded at around 460 nm in the case of DAPI and at 485 nm in the case of Hoechst dyes (Supplementary Fig. S3).

### The linearity and sensitivity of the developed assay

#### The linearity

**Figure 4:**
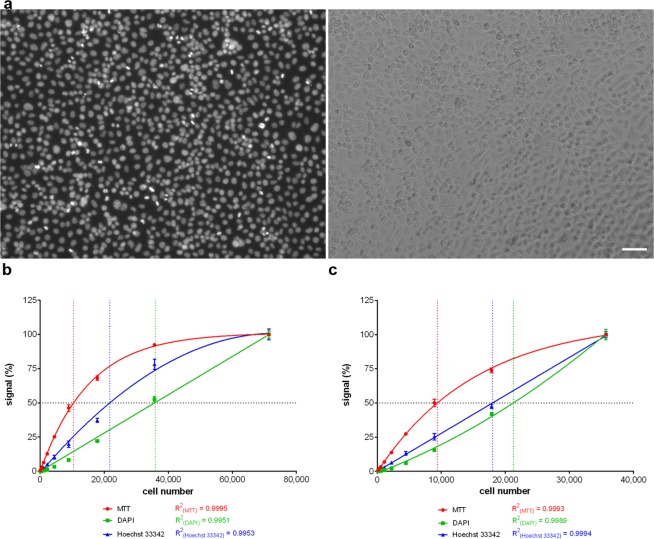
Dependence of the signal on the cell number, test of the linearity. **(a**) The microscopic images of the HeLa cells grown in the concentration of ca 71,000 cells per well of the 96-well plate. Cell nuclei labelled with DAPI (left) and the phase contrast image of the same cells (right) is shown. Bar – 50 µm. (**b**) The dependence of the signal provided by the developed approach using DAPI or Hoechst 33342 and by MTT assay on the number of HeLa cells is shown. The highest HeLa concentration was 71,000 cells/well of the 96-well plate. Serially twofold diluted cells were used. The signal is normalised to the signal measured in samples with the highest cell number which is equal to 100%. The black dotted line intersects the curves at the point corresponding to 50% of the maximal signal. The data are presented as the mean ± SD. (**c**) The dependence of the signal provided by the developed approach using DAPI or Hoechst 33342 and by MTT assay on the number of HeLa cells is shown. The highest HeLa concentration was 35,500 cells/well of the 96-well plate. Serially twofold diluted cells were used. The signal is normalised to the signal measured in samples with the highest cell number which is equal to 100%. The black dotted line intersects the curves at the point corresponding to 50% of the maximal signal. The data are presented as the mean ± SD.

We tested the linearity of the developed approach as well. Serially diluted HeLa cells or diploid fibroblasts IMR-90 were grown in a 96-well plate. The highest concentration (HC) of the cells was ca 71,000 HeLa cells or ca 80,000 IMR-90 cells per well. These concentrations correspond to an approximately 100% confluent cell population (Fig. 4a). The number of cells was evaluated either by image cytometry using CellProfiler software or by the manual counting. Eight images per well were acquired and evaluated.

We compared the linearity of the approach using DAPI or Hoechst 33342 staining with the approach based on the conversion of 3-(4,5-dimethylthiazol-2-yl)-2,5-diphenyltetrazolium bromide (MTT). From the graph of HeLa cells (Fig. 4b), it is evident that the highest linearity was provided by DAPI staining (linear regression, R^2^ > 0.9951), then by Hoechst 33342 staining (second-order polynomial regression, R^2^ > 0.9953) and the lowest by MTT (one-phase association regression, R^2^ > 0.9995). In this respect, half of the maximal signal corresponded to 50%, 30% or 15% of the number of cells in the case of DAPI, Hoechst 33342 and MTT, respectively.

If the maximal cell confluence decreased to 50% (35,500 cells per well), the highest linearity was provided by Hoechst 33342 dye (Fig. 4c, linear regression, R^2^ > 0.9994. DAPI and especially MTT provided lower linearity (Fig. 4c).

If the linear regression was used for the graph construction of all dyes, the R^2^ values were equal to 0.9951, 0.9335 and 0.6863 (HC = 71,000 HeLa cells; Supplementary Fig. S4a) or 0.9805, 0.9994 and 0.8866 (HC = 35,500 HeLa cells; Supplementary Fig. S4b) for DAPI, Hoechst 33342 and MTT, respectively. Similar results were obtained with the serially diluted human fibroblasts IMR-90 (HC = 80,000 IMR-90 cells; Supplementary Fig. S5a) or MRC-5.

The analysis of the linearity of the signal provided by Hoechst 33258 (Supplementary Fig. S5b) have shown that this dye provides lower linearity than Hoechst 33342. As this dye also provided a lower signal (Supplementary Fig. S1) and higher dependence on the content of RNA (Fig. 2), this dye is much less appropriate for cell quantification than Hoechst 33342.

We also tested re-staining the cells after the elution of the DNA dyes from the DNA. According to our results, this protocol provides similar data as was provided by the first staining and elution. On the other hand, it requires at least four washing steps with 20 mM Tris-HCl, pH 7, 150 mM NaCl after the first elution step. The total length of this washing should be at least 30 minutes. In addition, at least a 2-hour incubation with DNA dyes is necessary. This time should even be prolonged to 4 hours if highly confluent cell populations are used, otherwise, the cells with the higher confluence will contain a lower amount of the dye per cell than cells with lower confluence.

#### The sensitivity

**Figure 5:**
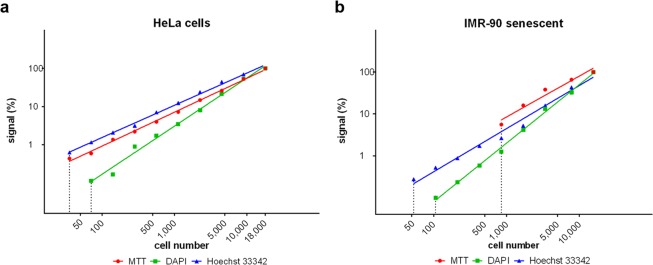
Dependence of the signal on the cell number, test of the sensitivity. **(a**) The sensitivity of the developed approach using either DAPI or Hoechst 33342 and MTT assay for HeLa cells is shown. The signal is normalised to the signal measured in samples with the highest cell number which is equal to 100%. The log scales of both dye concentrations and signal intensity are used. (**b**) The sensitivity of the developed approach using either DAPI or Hoechst 33342 and MTT assay for late-passage IMR-90 cells is shown. The signal is normalised to the signal measured in samples with the highest cell number which is equal to 100%. The log scales of both dye concentrations and signal intensity are used.

HeLa cells and late-passage IMR-90 human fibroblasts were seeded in 96-well plates in serial dilutions starting at 18,000 cells (HeLa cells) or 15,500 cells (IMR-90) per well. The cells were quantified using the developed approach or according to the standard protocol for MTT assay. The MTT assay and the developed approach based on Hoechst 33342 provided similar sensitivity in the case of HeLa cells which usually has a hypertriploid chromosome number^22,23^. The detection limit was ca 35 HeLa cells per well (Fig. 5a). In the case of DAPI, the detection limit was ca 70 cells per well (Fig. 5a). On the other hand, the sensitivity of the MTT assay was much lower if late-passage diploid fibroblasts were used. In this case, the detection limit was around 840 cells (Fig. 5b). If the developed approach was used, the detection limit was around 50 or 100 cells for Hoechst 33342 and DAPI staining, respectively (Fig. 5b). In the case of late-passage human diploid fibroblasts MRC-5, the detection limit was around 70, 140 and 560 cells for Hoechst 33342 assay, DAPI assay and MTT assay, respectively (Supplementary Fig. S6a).

### The use of the developed approach for cytotoxicity evaluation

**Figure 6:**
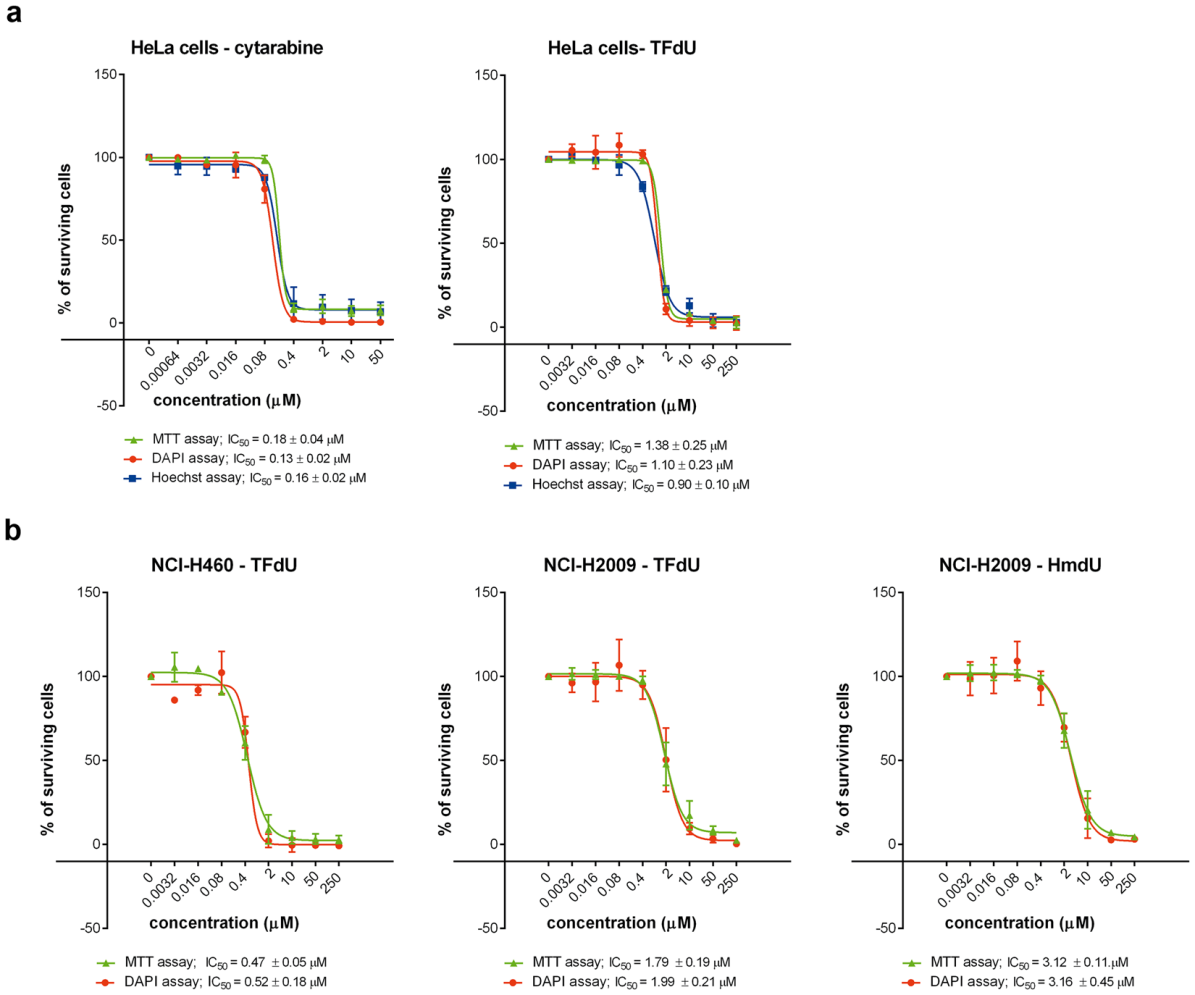
Cytotoxicity assay. (**a**) A comparison of the results of cytotoxicity evaluation of cytarabine and TFdU for HeLa cells provided by the developed approach based on DAPI or Hoechst 33342 or by MTT assay is shown. The data are presented as the mean ± SD. (**b**) A comparison of the results of cytotoxicity evaluation of TFdU for NCI-H460 and NCI-H2009 cells and HmdU for NCI-H2009 cells provided by the developed approach based on DAPI or by MTT assay is shown. The data are presented as the mean ± SD.

We also tested the use of the developed approach for the evaluation of the cytotoxicity of various substances. We cultivated cells seeded in the 96-well plates in a culture media containing serial five-fold dilutions of cytarabine or 5-trifluoromethyl-2′-deoxyuridine (TFdU) or 5-hydroxymethyl-2′-deoxyuridine (HmdU) for two population doubling times of a particular cell line. Then, we exchanged the medium for a fresh one and cultivated the cells for three population doubling times corresponding to the particular cell line. Next, we performed either the standard MTT assay or the developed assay. The signal was measured using the plate reader and evaluated. All the tested methods provided very similar IC_50_ values (Fig. 6).

### Simultaneous detection of cellular components and cell cycle analysis

As the samples are fixed, the developed method is compatible with the detection of various cellular components. In this respect, we successfully tested detection of the splicing factor SC35 and coilin – a marker of coiled bodies and the detection of DNA replication using 5-ethynyl-2′-deoxyuridine (EdU). If the protein detection was performed, the DAPI or Hoechst solution contained antibodies against the particular proteins. The EdU detection was performed before DAPI or Hoechst staining. The SC35, coilin or EdU signal was analysed before the application of washing solutions containing copper ions. As some proteins and/or antibodies requires e.g. formaldehyde fixation, the appropriate protocol has to be tested first. If the cell cycle analysis was performed, stained samples were washed and pictures for image cytometry were taken. Then, the DNA-bound dyes were eluted and the signal was measured (Supplementary Fig. S6b). The possibility to perform the detection of various components and cell cycle analysis simultaneously with the cell quantification allows various analyses to be conducted using the same cells and to address e.g. the effect of the cytotoxicity of various compounds in a more complex way.

## Discussion

Various assays for the measurement of cell proliferation are presently available. The most used approaches exploit two basic strategies. The first is based on the measurement of the enzyme activities. The second is based on the determination of the amount of nucleic acids. The enzyme activities in cell samples are most commonly measured by MTT, MTS, WST-1 and Alamar Blue.

Metabolic assays do not actually measure the cell number of viable cells in a culture or their growth, but rather a set of enzyme activities, that are related in various ways to the cellular metabolism^1^. The metabolic assays are highly sensitive approaches if highly metabolically active cell lines are used. On the other hand, its sensitivity is quickly lost if slowly grown cells or senescent cell lines are used^24^. In this respect, we showed that the sensitivity of MTT assay was much lower if late-passage diploid fibroblasts, probably corresponding to the senescent cells, were used. As the metabolic processes can be lowered at high cell densities, the metabolic assays are not suitable for assessing cell proliferation in such situations^1,10^. It is, therefore, not surprising that the linearity of the MTT assay was lower than that of the developed approach. Metabolic approaches also usually require longer times for their completion than the developed approach as the production of a sufficient amount of the coloured product may take several hours. The high variability between the different cell lines also results in the necessity of discovering the optimal time and concentration of the substrate. If longer incubation times are used, the eventual toxicity of the substrates should be taken into account as well^24^.

The developed approach does not depend on the cell line and is definitely shorter than 2 hours including the time necessary for the data collection. It can be also interrupted for at least 3 months (after fixation) or for at least 20 days (after elution). Another disadvantage of metabolic assays is the necessity to add the substrates under sterile conditions as cells are usually returned to the incubators. There is no need for sterility in the case of the developed approach.

The common approaches based on the measurement of the amount of nucleic acids usually derives its benefit from the fact that the binding of some molecules to nucleic acid results in a significant increase of their fluorescence. Such molecules include Hoechst 33258^25^, Hoechst 33342^25–27^, propidium iodide^28^, PicoGreen^29^ or CyQuant^11^. The enhancement of the fluorescence is dye specific. A 30-40-fold increase of the fluorescence was reported for Hoechst 33342 after its binding to DNA compared to the unbound dye^30,31^. The fluorescence of propidium iodide increases up to 30-fold^32^, Hoechst 33258 30-fold^31^ and DAPI 20-fold^31,33^. Much higher fluorescence enhancement was measured in the case of PicoGreen exhibiting more than a 1,000-fold increase of the fluorescence intensity^34^.

In this respect, we found that the presence of SDS results in around a 90-fold increase of the fluorescence of DAPI, a 700-fold increase in the case of Hoechst 333258 and around a 1,000-fold signal increase in the case of Hoechst 33342. However, the increase of the fluorescence intensity is not so important in the developed assay. Instead, the absolute fluorescence intensity plays the most important role as only the dye eluted from the cells is analysed. In this respect, Hoechst 33258 provided only 75% of the signal of Hoechst 33342, while DAPI provided only 45% of this signal.

The sensitivity of the above-mentioned assays differs substantially. While 100 cells can be revealed by PicoGreen^29^ and even less than 50 cells by CyQuant^11^, 1,000 cells is the detection limit of the assays based on propidium iodide or the previously described assays based on Hoechst dyes^29^. The sensitivity of the presented approach is between PicoGreen and CyQuant. On the other hand, the disadvantage of the CyQuant assay is the necessity to perform a freezing/thawing cycle by the freezing of samples at −70 °C followed by cell lysis for the maximum precision and linearity in wide ranges of cell numbers^11^. This protocol provides linear data from 50 to 50,000 cells. If higher concentration ranges need to be analysed (up to 250,000 cells), the concentration of the dye has to be increased^11^. Although a variant of the CyQuant approach based on cell permeabilisation without a freezing step can be used, it provides the linear data from 100 to 20,000 cells only. In this respect, the developed approach provides linear data from around 70 HeLa cells to the fully confluent cell population (ca 71,000 HeLa cells per well of a 96-well plate) without the need of repeated sample freezing/thawing and cell lysis if DAPI is used. Even higher sensitivity and linearity (35 HeLa cells or 50-70 human diploid fibroblasts per well of a 96-well plate) can be obtained if Hoechst 33342 is used. However, in the case of Hoechst 33342, the maximum number of cells should correspond to the around 35,500 cells per well of a 96-well plate (around 50% confluence).

In the case of the PicoGreen assay, it is also necessary to use cell lysis for maximum sensitivity^29^. The cell lysis excludes the simultaneous analysis of the cell cycle by image cytometry or simultaneous analysis of the cell population by immunocytochemical methods. In this respect, the method developed does not require cell lysis. As the approach is compatible with the commonly used ethanol fixation, both the cell cycle analysis using image cytometry or immunocytochemical detections can be performed before cell quantification. Moreover, DAPI and Hoechst staining is fully compatible with the commonly used fluorochromes as their excitation and emission spectra are sufficiently separated from the spectra of DAPI and Hoechst dyes. The cells fixed by formaldehyde can be also used during the developed assay. However, as the formaldehyde fixation provides approximately half of the signal obtained after ethanol fixation, it can result in lower sensitivity. Another advantage of the developed method is the possibility of repeated staining of the cells if necessary (e.g. if there is an error).

Concerning the cytotoxicity studies, the developed approach provided very similar data as the MTT assay despite the fact that that it provides much higher linearity. It is probably the result of the very steep decline of the survival of the cells if fivefold dilution of the tested substances is used. We suppose that the presented procedure can also be highly valuable if metabolically less active adherent cells are processed, and the low sensitivity of metabolic assays, therefore makes it impossible to obtain the relevant data.

As was the case with the other methods, the method developed provides relative data. After the calibration, e.g. by means of a haemocytometer or cameras and suitable software, it is possible to determine the absolute number of cells in the particular sample.

The described method of cell quantification is fast and thanks to the low material demands and the fact that the measurement takes place in another vessel than the one in which the cells are cultivated, it is suitable for processing high numbers of samples and/or large samples. Concerning the price of the test, the cost of the chemicals used for the processing of one 96-well plate is around 1.5 USD. Although the price of the black well plate increases the expenditures, it can be minimized to very low level, as it can be washed and used repeatedly. In this respect, the two washing steps with 0.2 M KOH or the washing buffer used for Hoechst dyes for 30 minutes followed by two washing steps in tap water and one washing step with deionized water provided similar results as a new black well plate.

## Conclusions

The developed method of cell quantification is based on the measurement of the DNA content. In contrast to commonly used methods based on the same strategy, it does not rely on the enhancement of the signal by binding the dye to nucleic acids. Instead, Hoechst or DAPI dyes bound to DNA are eluted using an elution buffer and the enhancement of the signal is performed in this buffer by SDS. The formation of micelles of SDS results in a high enhancement of the fluorescence of the used dyes (e.g. up to a 1,000-fold increase of the fluorescence intensity in the case of Hoechst 33342). The signal is high for at least 20 days at RT. The procedure can be also interrupted for several months after ethanol fixation. As there is no need for cell lysis, the cell cycle analysis using image cytometry or the detection of various cellular components can be performed before the elution step. For the samples with a cell density below 50% confluence, Hoechst 33342 should be used. It makes it possible to obtain high linearity and sensitivity. If the samples with cells at a higher density are used, DAPI should be the preferred choice. The method can be also used for cytotoxicity evaluation.

## Methods

### Cell culture

Human HeLa cells (cervix, adenocarcinoma; a gift from Dr. David Staněk, Institute of Molecular Genetics, Prague), NCI-H2009 (lung, adenocarcinoma), NCI-H460 (lung, carcinoma; both latter cell lines are a gift from Dr. Marián Hajdúch, Institute of Molecular and Translational Medicine, Olomouc), human diploid fibroblasts: IMR-90 cells (lung, ATCC, CCL-186) and MRC-5 cells (lung; a gift from Dr. Marián Hajdúch, Institute of Molecular and Translational Medicine, Olomouc) were used.

The HeLa cells, NCI-H2009 and NCI-H460 cells were cultivated in Dulbecco’s modified Eagle’s medium (DMEM) supplemented with 10% foetal bovine serum, 3.7 g/l of sodium bicarbonate and 50 µg/ml of gentamicin. The IMR-90 and MRC-5 cells were cultivated in Eagle’s minimum essential medium (EMEM) supplemented with 20% foetal bovine serum, 3.7 g/l of sodium bicarbonate and 50 µg/ml of gentamicin. The cells were cultivated in culture flasks or in a Petri dishes or in 96-well plates at 37 °C in a humidified atmosphere containing 5% CO_2_.

### The dependence of the signal on the cell number

For the testing of the linearity of the signal dependence on the cell number per well of the 96-well plate, the serially diluted cells (factor of dilution = 2) were seeded and incubated for 18 hours. Then, the plates were processed according to MTT assay protocol^35–37^ and the instructions of the MTT distributor (Thermo Fisher Scientific) or according to the developed approach. In the case of MTT, the freshly-prepared 1.1 mM MTT was added and the samples were incubated for 3 hours at 37 °C in a humidified atmosphere containing 5% CO_2_. The culture media were removed (except 10 µl) and 100 µl of DMSO was added to each well. The samples were incubated for 10 minutes at 37 °C and 500 rpm in the Thermomixer chamber. The absorbance was measured using a plate reader at 540 nm.

### Cytotoxicity evaluation

If the toxicity tests were performed, the cells were seeded at the density of 5 × 10^3^ cells per well in 96-well plates and incubated for 24 hours. Then, the modified nucleosides were added to the culture media. Serial fivefold dilutions of cytarabine starting at 0.00064 µM and ending at 50 µM and serial fivefold dilutions of TFdU or HmdU starting at 0.0032 µM and ending at 250 µM were used. The cells were further incubated for the time corresponding to the two population doubling times (HeLa cells and NCI-H460 – 48 hours, NCI-H2009 – 64 hours). Then, the culture media were exchanged for nucleoside-free media and the cells were incubated for an additional three population doubling times (HeLa cells and NCI-H460 – 72 hours, NCI-H2009 – 96 hours). Further, 1.1 mM MTT was added and the same protocol was used as in the case of the linearity testing or the plates were processed according to the developed approach.

### Fixation protocols

The cells were rinsed with 1× PBS and fixed with 70% ethanol or 100% methanol for 10 minutes or 30 minutes or 1 hour or overnight at −20 °C or 0 °C or at RT. In some cases, the culture medium was removed from the culture dish and the fixative was added directly to the cells without the previous washing with the buffer. In some experiments, the cells fixed in 70% ethanol were stored for 3 months at −20 °C.

When formaldehyde was used as fixative, the cells were rinsed with 1× PBS and 2% formaldehyde in 1× PBS was added to the samples for 10 minutes at RT. In this case, the cells were further permeabilised with 0.2% Triton X-100 in 1x PBS for 10 minutes.

In the case of air-drying fixation, the cells were rinsed with 1× PBS, the buffer was removed and the cells were air dried.

### Optimization of the SDS concentration, the effect of micelle formation and excitation and emission spectra measurement

The 1 µM solutions of DAPI or Hoechst dyes in water or in 20 mM phosphate buffer, pH 6 or pH 7 or pH 8 or in 20 mM Tris-HCl, pH 7 containing various concentrations of SDS or without SDS were prepared. The 0.1 ml of the prepared solutions was then transferred into the black 96-well plate and the fluorescence was measured using the plate reader. The buffered solutions of 1 µM DAPI or Hoechst dye 33342 containing 8%, 2%, 0.5%, 0.125% and 0.3125% SDS were used during the optimization of the SDS concentrations.

For the analysis of the effect of micelle formation on the signal intensity, a 1 µM solution of DAPI or Hoechst dyes in water containing 0.5%, 0.25%, 0.125%, 0.0625%, 0.03125%, 0.015625%, 0.0078125% or 0.00390625% SDS was prepared, then 0.1 ml of the solution was transferred into the black 96-well plate and the fluorescence was measured.

The excitation and the emission spectra of DAPI were measured using a 1 µM solution of DAPI in 20 mM Tris–HCl, pH 7 and 2% SDS. The excitation and the emission spectra of Hoechst 33342 and 33258 were measured using a 1 µM solution of Hoechst dyes in a 20 mM phosphate buffer, pH 7 and 2% SDS. For the emission spectra measurement, the excitation wavelength was 370 nm. For the excitation spectra measurement, the emission wavelength was 470 nm.

### Impact of RNase A

When the impact of RNase A was measured, the HeLa cells were fixed with 70% ethanol for 10 minutes at RT, air-dried and incubated with the solution of RNase A. The final concentration of RNase A was 0.1 mg/ml. The RNase A was diluted in 1× PBS. In the control samples, 1× PBS without RNase A was added. The samples were incubated 60 minutes at 37 °C and 300 rpm. Then, the samples were processed according to the developed protocol.

### Cell counting

Serially diluted cells cultivated in 96-well plates were fixed with 70% ethanol for 10 minutes at RT. The samples were air-dried and stained with the solution of 3 µM DAPI in 20 mM Tris–HCl, pH 7 and 150 mM NaCl. Eight images per well were acquired and evaluated. The number of cells was evaluated either by image cytometry using CellProfiler software^38,39^ or by manual counting.

### Protein detection, the detection of DNA replication and cell cycle analysis

Mouse anti-SC35 antibody (1∶250, Abcam, ab11826) or mouse anti-coilin antibody (1∶50, Abcam, ab87913) were used for the protein detections^40^. In this case, a particular antibody was added to staining solution. After the washing and rinsing step, proteins were detected using Alexa Fluor 488 anti-mouse antibody (1∶100) diluted in 25 mM Tris-HCl, pH 7.4, 150 mM NaCl. Protein detection was evaluated prior to the elution step.

DNA replication was detected using EdU. The cells were incubated with 10 µM EdU for 30 minutes prior to the ethanol fixation. Then, EdU was detected using a click reaction^36^ followed by the developed approach. The EdU signal was analysed prior to the elution step.

If the cell cycle analysis was performed, the image cytometry was performed prior to the elution step. The cell cycle analysis by image cytometry was performed according to^37^.

### Data acquisition and evaluation

In the case of black 96-well plates, the signal was measured using the Infinite 200 Pro Plate Reader (Tecan).

If the image cytometry or manual counting was used for the sample evaluation, the images were obtained by an Olympus IX81 microscope (objective: UPLFLN 10 × NA 0.3) equipped with a Hamamatsu ORCA II camera (a resolution of 1344 × 1024 pixels) using Cell ∧ R acquisition software.

The data were analysed using CellProfiler and Microsoft Excel and the final graphs were made in GraphPad Prism 6. The graphs were constructed using the following functions:

1. Standard four parameter logistic nonlinear regression was used in the case of the analysis of the standard cytotoxic test by MTT assay or by the developed approach.
2. One-phase association was used for the analysis of the signal dependence on the number of HeLa cells in the case of MTT assay.
3. Linear regression or second order polynomial (quadratic) regression was used for the analysis of the signal dependence on the concentration of the DNA dyes diluted in 2% SDS, the signal dependence on the number of HeLa cells, IMR-90 cells and MRC-5 in the case of Hoechst 33342, DAPI and MTT assay

All the performed experiments were done in three independent replicates. The data are presented as the mean values ± standard deviation (SD).

The Fig. 1 was done using Rhinoceros 5 and Adobe Photoshop CS4 software.

## Supplementary information

Supplementary information

## Data Availability

All data generated or analysed during this study are included in this published article (and its Supplementary Information files).
