# Supplementary material for: Quantification of fixed adherent cells using a strong enhancer of the fluorescence of DNA dyes
Source: Sci Rep. 2019 Jun 18;9:8701. doi: 10.1038/s41598-019-45217-9 (PMC6581942; doi:10.1038/s41598-019-45217-9)
Supplement: Supplementary file 1 — Supplementary information [file 41598_2019_45217_MOESM1_ESM.pdf]

Supplementary Information

Quantification of fixed adherent cells using a strong enhancer of the fluorescence of DNA dyes

Anna Ligasová* and Karel Koberna*

Institute of Molecular and Translational Medicine, Faculty of Medicine and Dentistry, Palacký University in Olomouc, Olomouc, 779 00, Czech Republic

*Correspondence: anna.ligasova@upol.cz (A.L.); karel.koberna@upol.cz (K.K.)

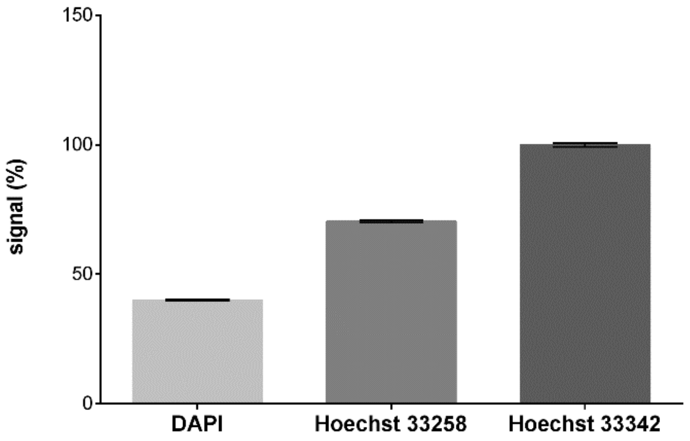

Supplementary Figure S1. Comparison of the signal intensities
The signal intensities for particular DNA dyes is shown. The fluorescence intensity of the dyes in 2% SDS and 20 mM phosphate buffer, pH 7 (Hoechst dyes) or 20 mM Tris-HCl buffer, pH 7 (DAPI) was measured. The signal is normalised to the signal measured in the samples with Hoechst 33342 which is equal to 100%. The data are presented as mean ± SD.

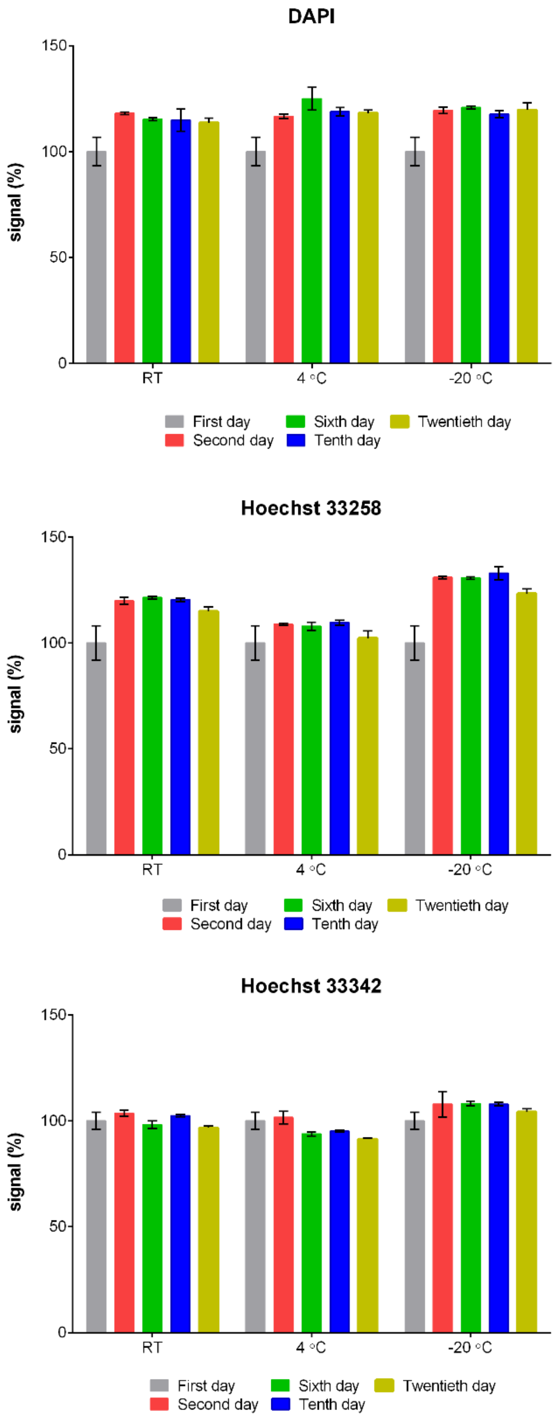

Supplementary Figure S2. The stability of the signal

The stability of the DAPI, Hoechst 33258 and Hoechst 33342 signal measured at various temperatures for 20 days is shown. HeLa cells grown in Petri dishes were processed using the developed approach and the eluted dyes were stored at RT or at 4°C or at -20°C. The signal was measured on the day of experiment (the first day), on the second day, on the sixth day, on the tenth day and on the twentieth day. All samples were warmed up to RT before the measurement. The signal is normalised to the signal measured in the samples measured on the day of experiment (the first day) which is equal to 100%. The data are presented as the mean ± SD.

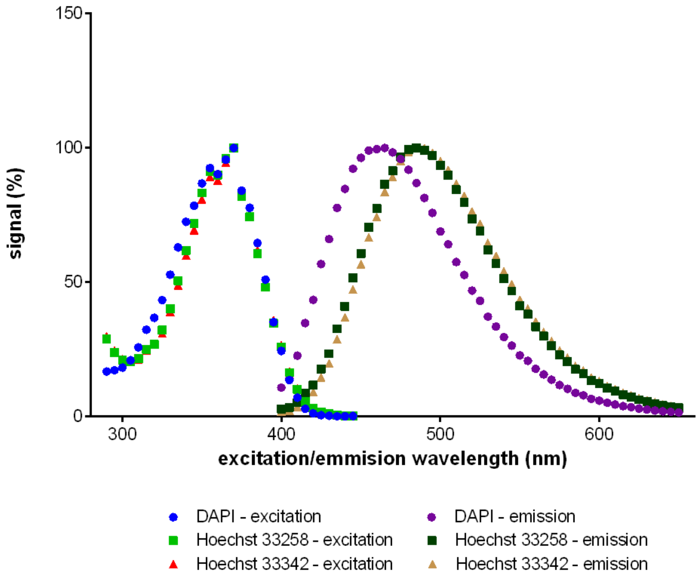

Supplementary Figure S3. Excitation and emission spectra of DNA dyes

The excitation and emission spectra of DAPI in 20 mM Tris-HCl buffer, pH 7 with 2% SDS and Hoechst 33342 and Hoechst 33258 in 20 mM phosphate buffer, pH 7 with 2% SDS are shown. The excitation was measured every 5 nm from 290 nm to 445 nm (emission = 470 nm), the emission was measured every 5 nm from 400 nm to 650 nm (excitation= 370 nm).

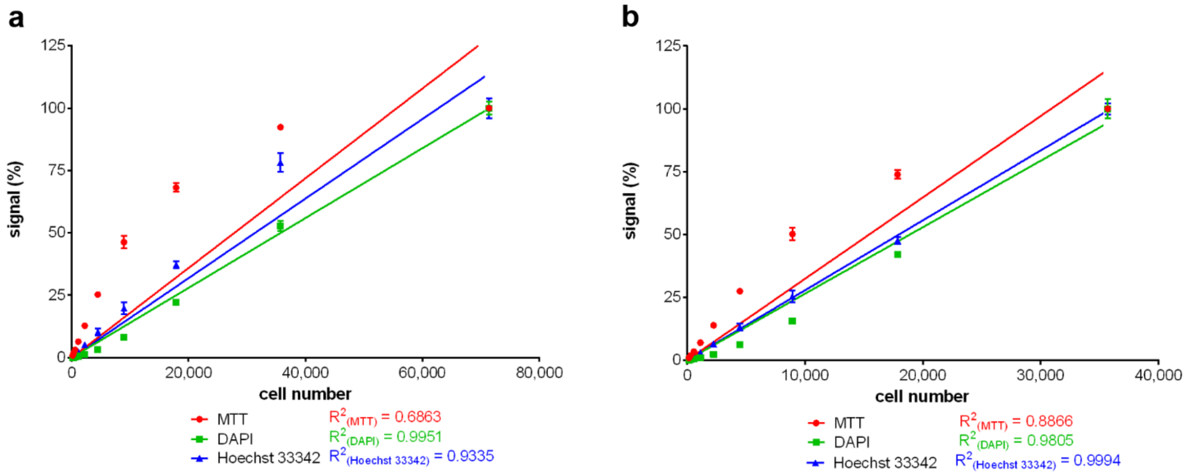

Supplementary Figure S4. The dependence of the signal on the cell number

a) The dependence of the signal on the number of HeLa cells is shown for the developed approach using DAPI or Hoechst 33342 or for MTT assay. The highest cell concentration was 71,000 cells/well of the 96-well plate. Serially twofold diluted cells were used. The number of cells was checked by image cytometry or manual counting in microscopy images. The signal is normalised to the signal measured in the samples with the highest cell number which is equal to 100%. Linear regression was used to construct the lines. The data are presented as the mean ± SD.

b) The dependence of the signal on the number of HeLa cells is shown for the developed approach using DAPI or Hoechst 33342 or for MTT assay. The highest cell concentration was 35,000 cells/well of the 96-well plate. Serially twofold diluted cells were used. The number of cells was checked by image cytometry or manual counting in microscopy images. The signal is normalised to the signal measured in the samples with the highest cell number which is equal to 100%. Linear regression was used to construct the lines. The data are presented as the mean ± SD.

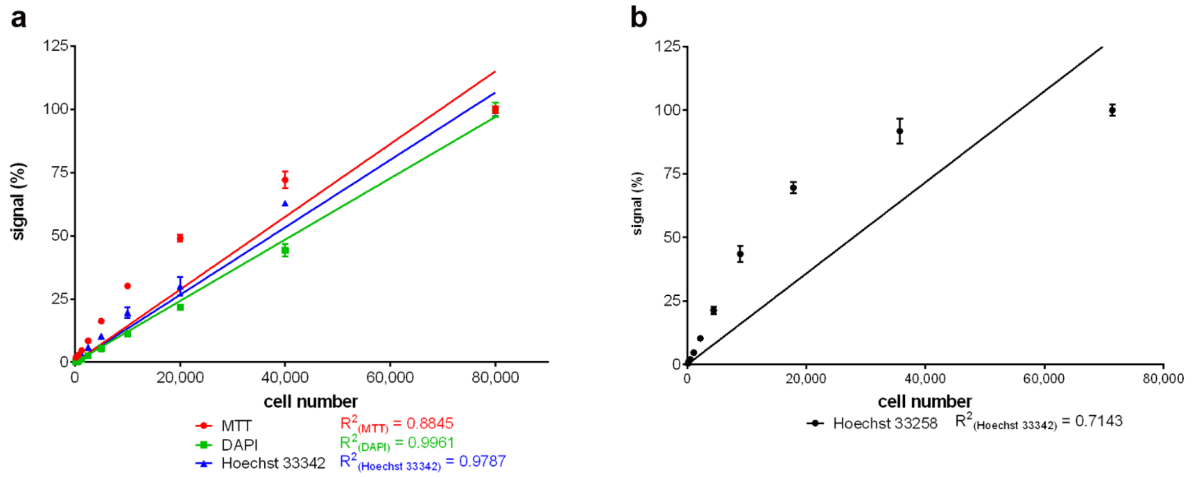

Supplementary Figure S5. The dependence of the signal on the cell number

a) The dependence of the signal on the number of IMR-90 cells is shown for the developed approach using DAPI or Hoechst 33342 or for MTT assay. The highest cell concentration was 80,000 cells/well of the 96-well plate. Serially twofold diluted cells were used. The number of cells was checked by image cytometry or manual counting in microscopy images. The signal is normalised to the signal measured in the samples with the highest cell number which is equal to 100%. Linear regression was used to construct the lines. The data are presented as the mean ± SD.

b) The dependence of the signal on the number of HeLa cells is shown for the developed approach using Hoechst 33258. The highest cell concentration was 71,000 cells/well of the 96-well plate. Serially twofold diluted cells were used. The number of cells was checked by image cytometry or manual counting in microscopy images. The signal is normalised to the signal measured in the samples with the highest cell number which is equal to 100%. Linear regression was used to construct the line. The data are presented as the mean ± SD.

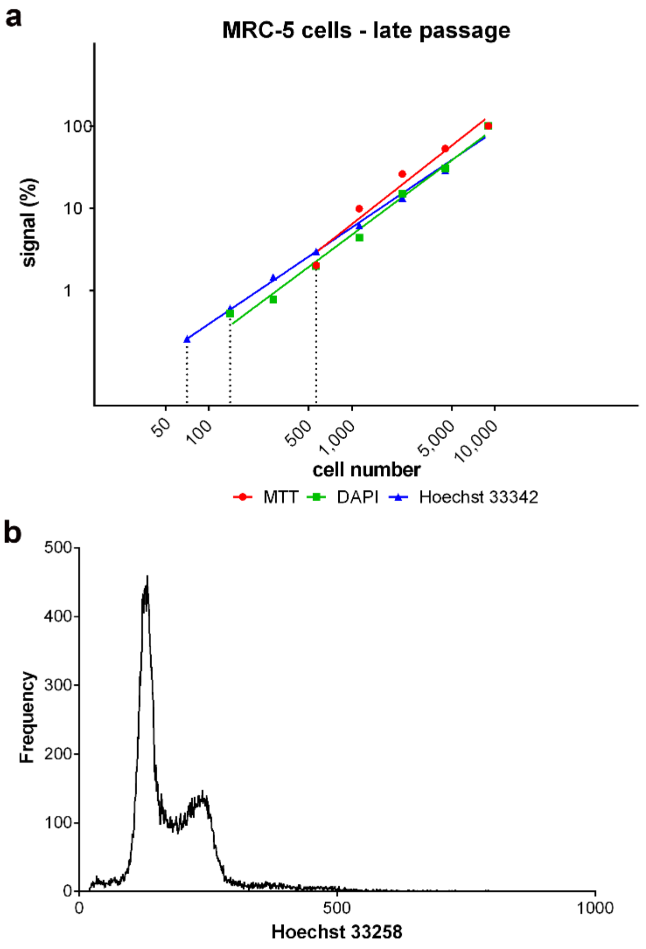

Supplementary Figure S6. The sensitivity of the approach and cell cycle analysis

A) The sensitivity of the developed approach using either DAPI or Hoechst 33342 and MTT assay in late-passage MRC-5 cells is shown. The signal is normalised to the signal measured in samples with the highest cell number which is equal to 100%. The log scales of both dye concentrations and signal intensity are used.

B) The simultaneous cell cycle analysis with the developed approach is shown. HeLa cells were stained, washed and pictures for image cytometry were taken. Then, the DNA-bound dyes were eluted and the signal was measured. The cell cycle was analysed with CellProfiler and the data were evaluated using Microsoft Office and GraphPad Prism software.
